# Food Security Experiences of Aboriginal and Torres Strait Islander Families with Young Children in An Urban Setting: Influencing Factors and Coping Strategies

**DOI:** 10.3390/ijerph15122649

**Published:** 2018-11-26

**Authors:** Leisa McCarthy, Anne B. Chang, Julie Brimblecombe

**Affiliations:** 1 Menzies School of Health Research, 0870 Darwin, Australia; anne.chang@menzies.edu.au (A.B.C.); julie.brimblecombe@monash.edu.au (J.B.); 2 Department of Respiratory Medicine, Queensland Children’s Hospital, 4101 Brisbane, Australia; 3 Children’s Centre for Health Research, Queensland University of Technology; 4101 Brisbane, Australia; 4 Department of Nutrition, Dietetics and Food, School of Clinical Sciences, Monash University, 3168 Melbourne, Australia

**Keywords:** food security, food insecurity, Aboriginal and Torres Strait Islander population, children, urban, experiences, coping strategies

## Abstract

Evidence on Aboriginal and Torres Strait Islander peoples’ food security experiences and coping strategies used when food insecurity occurs is limited. Such evidence is important to inform policies that can reduce the consequences of food insecurity. This study investigated factors perceived by Aboriginal and Torres Strait Islander families with young children to influence household food security, and coping strategies used, in an urban setting. A qualitative research inductive approach was used. Data were collected through an iterative process of inquiry through initial interviews with 30 primary care-givers, followed by in-depth interviews with six participants to further explore emerging themes. Major topics explored were: influencing factors, food insecurity experiences, impact on food selection, and coping strategies. Food affordability relating to income and living expenses was a major barrier to a healthy diet with large household bills impacting food choice and meal quality. Access to family support was the main reported coping strategy. Food insecurity is experienced by Aboriginal and Torres Strait Islander families, it is largely intermittent occurring especially when large household bills are due for payment. Family support provides an essential safety net and the implications of this are important to consider in public policy to address food insecurity.

## 1. Introduction

Food security is “access by all people, at all times to sufficient food for an active and healthy life. Food security includes at a minimum: the ready availability of nutritionally adequate and safe foods, and an assured ability to acquire acceptable foods in socially acceptable ways” [1] (p. 337). Irrespective of a country’s affluence status, some population groups within high income countries experience food insecurity and varying degrees of hunger. For these groups, strategies to overcome or alleviate food insecurity have been employed, but most measures are thought to be short-lived and a ‘stop gap’ to temporarily relieve problems [2,3,4,5,6,7,8,9,10,11]. Evidence that can inform longer-term solutions are required. Availability of such evidence is important to inform possible practice and policy interventions.

However, literature about people’s experiences with household food insecurity is limited, particularly within Indigenous populations of affluent countries, the group most at risk of household food insecurity and poor health [2,7,12,13,14]. Among families with young children in the United States and Canada, studies reported that although families access food assistance programs, food shortages and hunger are still experienced [2,3,4,5,6,7,8,9,10,11]. A study in six Inuit communities of Nunavut, Canada, focused on the availability and accessibility of traditional and market foods (i.e., foods purchased from a shop), found inconsistencies between perceived food security status and experiences in obtaining enough food to eat [7]. In contrast, another study undertaken with a Nunavut Inuit population, found participants who reported food insecurity also reported regular use of community food programs to assist with alleviating hunger [2]. The variance in results likely reflects the different sampling frame of these studies, as one [7] recruited from the broader community and the other [2] through food assistance programs [2,7].

To mitigate household food insecurity, coping strategies (i.e., the mechanisms families have in place to cope with food and money problems) are used [4,5,10,11,15]. A Canadian Quebec-based study described several coping strategies used by participants to overcome household food insecurity: adults reduced size of meals or forwent food so children could eat; modified lifestyle (e.g., forgoing purchases of less essential items and payment of bills to free up money for food); purchased sale item foods and foods close to use by date; and visited a food bank when desperate [10]. An Australian study undertaken in South Western Sydney investigated coping strategies [15]. The most frequent responses of nine coping strategies to select from were, cutting down on the variety of household foods (59.1%); a parent or guardian skipping meals or eating less (58.8%); and putting off paying bills (57.4%) [15]. Other coping strategies reported (among a multicultural group of 90 food pantry users in Washington, USA) include using leftover food, cooking food in bulk and freezing food for later use [11]; and among Latino immigrant families in North Carolina, USA, limiting food purchases considered expensive, e.g., meats and fruits and; shopping for specials and bulk-buying [5].

To alleviate household food insecurity, social support systems are important and include assistance from food programs, food charity organisations, faith communities, neighbours and friends [2,3,4,5,6,7,8,9,10,11,13]. Extended family as a social support system is also important [2,3,4,8,9,11] One study reported support from friends and neighbours as a main coping mechanism in response to food insecurity [5]. Within an Inuit population, living with family as a temporary coping strategy until housing was obtained was identified [2]. Another study, highlighted reciprocity where young mothers would rely on family members for food assistance and then ‘return the favour’ when other family members experienced difficulties [4].

Among Aboriginal and Torres Strait Islander peoples, the concept of reciprocity is an important component of the social connectedness [16]. This cultural sharing practice is important in maintaining and reinforcing individual and group social bonds and imparting knowledge about good food as related to balance—life, resources, food, knowledge [16,17]. Studies undertaken in Inuit populations have also noted reciprocity has a place in maintaining and reinforcing family and broader community relationship obligations as well as cultural identity and practice [2,7]. ‘Cultural sharing’ and ‘sharing networks’ ensure that excess traditional food is provided to the more vulnerable members of the community who cannot obtain these foods [2,7]. Identified in these studies, was that money or other services were exchanged for traditional foods to keep with continuing cultural practices for those who could not hunt [2,7]. Similarly, in a study undertaken with Latino Immigrant Families in North Carolina, USA, money was sent home to families to support food security. This action was justifiable from the belief family back home were in a worse situation to their own and a cultural obligation to look after ones’ parents [8].

Despite the importance of knowing coping mechanisms used when household food insecurity occurs within families, there is little to no such data among Aboriginal and Torres Strait Islander people. Obtaining such data provides knowledge on enablers that can inform policies to enhance existing coping strategies and support families. Therefore, this qualitative study explores the experiences of household food insecurity and coping strategies used among Aboriginal and Torres Strait Islander families residing in Darwin and Palmerston, two cities of remote Northern Australia.

## 2. Materials and Methods

A qualitative research inductive approach was used, where data collection involved an iterative process of inquiry through initial interviews and subsequent in-depth interviews. All interviews were undertaken by the primary author, an Aboriginal woman and nutritionist. An iterative process of data collection and analysis was undertaken. Thematic analysis was applied. The inductive method used was not confined to existing theoretical frameworks [18] or pre-determined categories and allowed for the creation of categories or codes during data collection to arise. These codes were then combined into themes which were mapped to reveal relationships between them. This process of qualitative data collection and analysis makes it particularly suitable to exploring an under-investigated study area.

This qualitative study was part of a larger study that aimed to investigate food security among families with use of a modified study version of the United States Department of Agriculture 18-item Household Food Security Module (mUS 18-item Module). It was during the administration of the mUS 18-item Module, that the initial interviews occurred and were initiated by the participants.

Human Research Ethics approval was obtained from the Human Research Ethics Committee of the Northern Territory Department of Health and Menzies School of Health Research and the Aboriginal Sub Ethics Committee. HREC File Reference Number 09/06.

### 2.1. Setting

The 2016 Australian Bureau of Statistics Census data estimated Aboriginal and Torres Strait Islander people comprised 3.3% of the Australian population (http://www.abs.gov.au/ausstats/abs@.nsf/mf/3238.0.55.001. 3238.0.55.001—Estimates of Aboriginal and Torres Strait Islander Australians, June 2016. LATEST ISSUE Released at 11:30AM (CANBERRA TIME) 31/08/2018). Darwin is the Northern Territory capital and Palmerston a nearby satellite city. Study data were collected for a period of 7 months between April 2009 and February 2010. At the time of the study population separations for Darwin and Palmerston were unavailable. Therefore, the combined total population of Darwin and Palmerston was 98,152 residents [19], with Aboriginal and Torres Strait Islander peoples comprising 7.5% of the total Palmerston population and 9.4% of the Darwin population [19]. The aim of this study was to investigate the food insecurity experiences of Aboriginal and Torres Strait Islander families in these two main centres of the Northern Territory.

### 2.2. Sampling

The primary author recruited potential participants through child health clinics in local health services’, comprising two Aboriginal health services and two Government health services. A local Aboriginal woman was employed to assist with recruitment at one of the health service sites. Participants were also recruited from the broader community through the assistance of an Aboriginal Research Officer who had extensive networks with Aboriginal families in both Darwin and Palmerston. Convenience sampling through the local health services and known networks was used.

### 2.3. Participant Recruitment

The inclusion criteria were: care giver of a young Aboriginal and/or Torres Strait Islander child (aged 6 months-4 years); resided in Darwin or Palmerston for ≥12 months; and the child did not have a medical condition requiring food or nutrition supplements. A set of predetermined participant inclusion criteria were developed prior to study commencement with input from the health services during the consultation phase. During recruitment, informed consent was obtained from the eligible child’s care-giver to participate in the whole study including qualitative interviews. Recruitment continued until 30 participants had completed the mUS 18-item Module, used to define the presence/absence of food insecurity. As the aim of the larger study was to test the reliability and face validity of the mUS 18-item Module among urban residing Aboriginal and Torres Strait Islander families with children 0.5 to 4 years, 30 participants were deemed a suitable number for this purpose.

### 2.4. Initial Discussions

Initial discussions, prompted through completion of the mUS 18-item Module, lasted from 30 min to three hours. Notes were taken (audio-recordings were not used) and read back to the participant to confirm the information and typed as a word document. Following each interview, the primary author coded text relating to food security experiences and coping strategies. These codes were explored in subsequent interviews. This iterative process continued until all initial interviews were complete. These codes informed the interview guide for the in-depth interviews (Appendix A).

### 2.5. In-Depth Interviews

In-depth interviews were audio recorded and notes taken. We purposively invited participants based on gender and identified age groups to reflect the overall study sample. The recordings were transcribed and the transcription read back to the participant either in person or over the telephone to verify the interview content and clarify any queries. Any adjustments or further data collected were agreed to, verified and included. The interview, transcription and analysis process continued iteratively until data saturation was reached i.e., when no new information or new themes emerged [20,21]. To achieve this, six participants were interviewed. Coding of all transcripts was undertaken firstly, by the primary author and then separately by the senior author. A set of themes identified initially by the primary author, was agreed on.

## 3. Results

Thirty care givers were recruited and engaged in initial discussions. Table 1 shows, the majority were female, Aboriginal and their age ranged between 17 and 58 years. Over half of the participants had partners (married or de facto relationship). The six participants were representative of the main sample in gender and age (Table 1).

### 3.1. Findings

**Figure 1:**
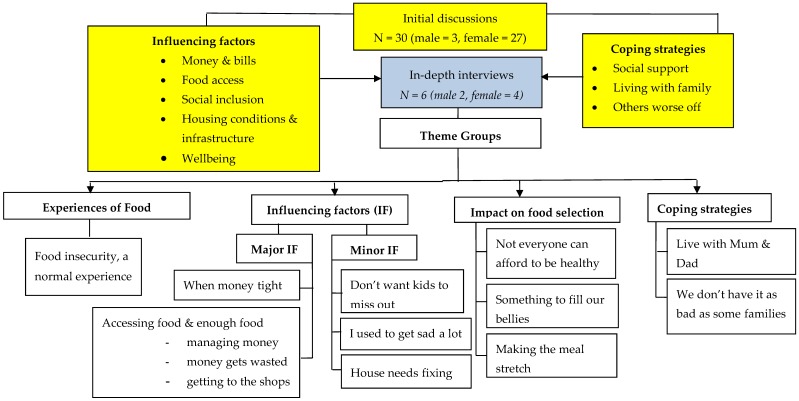
Overview of significant Qualitative Findings.

As shown in Figure 1, themes identified from the initial interviews were grouped according to (i) factors influencing food security and (ii) coping strategies. These were explored further in the in-depth interviews with the following themes identified: *(i) Experiences of Food Insecurity; (ii) Influencing Factors; (iii) Impact on food selection; and (iv) Coping Strategies*. Themes relating to influencing factors are presented as major or minor influencing factors and determined both by the number of participants referring to these themes and whether featured prominently in their responses. (Figure 1).

### 3.2. Experiences of Food Insecurity

#### Food Insecurity, a Normal Experience

Participants did not initially identify with being food insecure, although many of their experiences indicated otherwise. Whilst completing the mUS 18-item Module participants shared their own and others’ experiences with not having enough food, money or both and described this situation as common among themselves and/or people close to them. The experiences of food insecurity as told by participants implied that food insecurity was seen by most as a ‘normal’ experience and was also considered ‘the norm’ within their close social interactions:

There’s not enough money, full stop, to pay for food, to last from payday to payday.

(Aboriginal mother with four children, aged 34 years and partnered)

Another participant shared his observations when out shopping:

…I’ve seen people put food back. Put things back because they can’t get [afford] that. Or take a milk bottle back to get a smaller bottle of milk. Yes, you do see it around.

(Aboriginal and Torres Strait Islander father of two children, aged 36 years and partnered)

### 3.3. Influencing Factors

#### 3.3.1. Major Influencing Factors

These influencing factors were comprised of the sub-themes “*when money is tight*” and “*accessing food and enough food*”.

##### When Money is Tight

Many participants shared that they prioritised essentials when money was tight. Bills, such as quarterly electricity bills, were a main reason for making “money tight” and this impacted on the amount and type of food purchased. For some (*n* = 9), this situation was intermittent and occurred only when larger bills had to be paid or if there was a temporary change in household income. Whereas others (*n* = 11) described this as an everyday phenomenon. Four of these 11 participants mentioned not having enough money at all times due to an inadequate income. To ensure enough food for the family when ‘money was tight’ less expensive foods were chosen, such as highly processed foods of lower nutrient quality or cheaper brand options. For example, with fruit and vegetables, some participants purchased these in lesser quantity, did not buy at all, or purchased cheaper processed versions instead of fresh. Approximately half of the participants’ spoke of choosing less expensive foods to ensure children had something to eat at each meal when ‘money was tight’. This situation was not limited to participants who were recipients of Centrelink (The Centrelink Master Program is one of the Master Programs of the Australian Government Department of Human Services (Australia). The majority of Centrelink’s services are the disbursement of social security payments (Source: Wikipedia site *en.wikipedia.org/wiki/Centrelink.*)), as some participants in paid employment, or who had partners in paid employment, also experienced this phenomenon. One Aboriginal participant shared that despite both her and her husband having paid employment, they still sometimes experienced difficulties:

Sometimes we have to be tight [with money] when the big bills (electricity, car repayments) come in and choose less expensive foods to buy.

(Aboriginal mother of three children, 25 years and partnered)

This experience was echoed by an Aboriginal man, who as the sole income earner cut back on what he termed ‘luxury items’ such as snack foods, sweet drinks or desserts, when ‘money was tight’.

Don’t have real problems with food [having enough to eat] or with money. Only time may have to get tight with the budget is when the big bills come in. This just means cutting back on luxury items.

(Aboriginal father of one child, 38 years and partnered)

This participant also revealed other measures used to immediately relieve a “money tight” situation and ensure adequate food, although this measure had greater cost implications:

Often the bill would come in and we would go and do a shop and then make that shop stretch to the next pay to pay the bill. I would consider putting food in people’s guts (stomachs) more important than paying bills. If you don’t pay the bill on time, there’s a late fee $30, $40 dollars. Might as well pay it late, that’s how I would look at it.

(Aboriginal father of one child, 38 years, partnered)

Of the 30 participants, the sole income for nine were Centrelink payments and four of these individuals were being income managed (Income Management is an Australian Government initiative to assist individuals receiving Centrelink social security payments in managing money to meet essential household needs and expenses, and learn to better manage finances in the long term. (Source: http://www.humanservices.gov.au/customer/services/centrelink/income-management)). Three of the four participants considered these payments as inadequate in meeting their households’ basic living costs. A participant told of her experience in being income managed. She viewed income management as good for families who needed help with budgeting. In her case however it was not helpful as she ‘looked after her children properly’ and the payment was not enough to feed her family:

*I have three boys and you know, they eat a lot. One loaf of bread eaten for breakfast! I don’t think we get enough money and I can’t pay for all the food from my basic card Basic card (similar to a bank key card. A portion of income managed individuals’ payments are deposited into a basic card to purchase food and other essential household items only. (Source: http://www.humanservices.gov.au/customer/services/centrelink/income- management)*.

(Aboriginal mother of four children, 34 years and partnered)

Another participant, who stated that she had money problems all the time, worked full-time and had a regular income, but her partner had been having problems with securing permanent full-time employment:

I work full-time, but don’t get paid much. My partner works when he gets work and we also rely on government money [Centrelink payments]. The money that we do get seems to just cover the rent, food and basic necessities. Rent and food are expensive in Darwin. We also have a car to run and that’s also expensive.

(non-Indigenous mother of four children, 30 years and partnered)

Some participants shared that they experienced money problems temporarily due to a sudden change in their income, such as irregular timing in child maintenance payments:

Sometimes they [ex-partners] don’t pay [child maintenance] regularly and that throws us out with budgeting for the fortnight. I don’t think they [ex-partners] understand how hard it makes things sometimes.

(Aboriginal and Torres Strait Islander mother of three children, 38 years, single)

Other participants spoke of full-time employment and being paid well as an enabler of food security. An Aboriginal woman who was a full-time student expressed that her husband was in secure full-time employment, earned a “good wage” that was able to meet the family’s needs:

If my husband wasn’t on a good wage and he didn’t earn enough to cover the bills and other expenses, we would definitely be struggling.

(Aboriginal mother of one child, 27 years and partnered)

Nineteen of the 30 participants identified as food secure (63.3%), raised concerns about the rising cost of living and how this would impact on their families in the future. In particular, a man of both Aboriginal and Torres Strait Islander heritage spoke of not having money issues but mentioned the rising cost of living and the impact this could have on his household:

…it’s becoming very expensive. Everything has just gone up…and not just food prices. It’s electricity, phone, fuel [for car], the cost of living in general has gone up a lot. You know, we [participant and his wife] are aware of how difficult it could be if one of us lost our job. And we don’t have much in savings. And if there is an economic downturn that affects us, that’s why we’re trying to pay off as much of our mortgage now just to make sure that we have a buffer.

(Aboriginal and Torres Strait Islander father of two children, 36 years, partnered)

##### Accessing Food and Enough Food

This sub-theme encompassed findings relevant to budgeting and managing money; misuse of money; and food access.

###### Managing Money

At least five participants spoke of how important it is to budget or “manage money” to ensure enough money for food and bills. An Aboriginal woman shared her experience:

…I’ve always planned a budget to include extras to make sure money for additional expenses such as car maintenance, power bills, etc. Though, power bills have gone up. Not because we’re using more power, just the cost of power. Other things (essential items) are going up as well. You know, price of food, petrol, rent. So much pressure on families just to live. In our budget we always make sure the rent and bills are paid and there’s money for food. You know, the kids come first. Make sure they’re clothed, fed, school fees paid. Sometimes I may need new clothing, shoes, or whatever, but will go without to make sure the kids have what they need. Just make sure I have what I need budgeted for and save for it.

(Aboriginal mother with three children, 25 years and partnered)

For some though, budgeting did not always prevent “money being tight”:

…Sometimes things [budget] blows out and I think I mentioned it before. One month you might get your electricity bill and that. Plus, we have child care fees and that’s a big chunk out of that as well.

(Aboriginal and Torres Strait Islander father of two children, 36 years and partnered)

Four participants were income managed by Centrelink and had a portion of their income automatically quarantined for food and other household essentials accessed through use of a Basics card. There were mixed experiences with this system and two mentioned post-introduction of income management that money problems still occurred, whilst another two experienced improvements:

It’s ok. I don’t get much humbug (Humbug is a term predominantly used by Indigenous Australians in a way that means ‘to pester’, as in being pestered (humbugged) by someone for money) now [since introduction of basics card] for money and have enough money for food.

(Aboriginal grandmother, carer of 10 grandchildren, 44 years, widowed)

###### Money Gets Wasted

Although participants referred to their own struggles with managing money to meet family needs, many participants expressed that there were families in worse situations than themselves, particularly when anti-social behaviour such as gambling, excessive alcohol and illicit drug use were involved. Participants defined anti-social behaviour as that of ‘social problems’ and associated these with food and money problems:

…there are also problems with drinking [alcohol] and gambling. It makes me wonder sometimes, when people say they have no money to pay bills or buy food. They smoke [cigarettes], drink [alcohol] and gamble and don’t seem to understand this causes problems. When you have limited money, need to be smart about how to use it.

(Aboriginal and Torres Strait Islander mother of three, 38 years and single)

“Other families find it hard too [money problems]. That’s why some people sell drugs. Need more money. … have problems with gambling and drinking [alcohol]. Maybe drugs. A lot of money gets wasted. Make me sorry for the kids”.

(Aboriginal and Torres Strait Islander mother of five, 29 years, partnered)

Food security for some participants’ households were directly affected by others’ social problems:

My brother is bad. All he wants to do is drink grog [alcohol]. Then he gets hungry and comes here. Eats all my kids’ tucker [food]. He takes money from me and Nanna. Other people after him cos’ he steal grog [alcohol] from them.

(Aboriginal mother of two, 25 years and partnered)

###### Getting to the Shops

This sub-theme covered the ability to access food (shops) and reliable transport. Eleven participants mentioned their experience with accessing shops and how this impacted on food purchasing as well as seeking out food specials and bargains. Having access to supermarkets was considered by most participants as important to obtain affordable food items. Supermarkets were considered as cheaper and offering a wider variety of goods when compared to the smaller convenience type stores. During the study period a major supermarket chain outlet accessed by a quarter of the participants closed. The only other food outlet option locally available to these participants and within walking distance was the service station (a service station is a motor vehicle fuel outlet and often provides a small range of grocery items, including bread, milk, juice and a few dry goods lines.) which had only a small range of goods and was expensive. The impact of the supermarket closure on food security was expressed by an Aboriginal and Torres Strait Islander woman:

[I] find it hard with shopping since local supermarket closed. Shopping Centre not within walking distance but was a short drive from my house and [I] relied on a lift or taxi that didn’t cost very much. Now [I] have to pay more for taxis, as [I] travel further to go shopping.

(Aboriginal and Torres Strait Islander mother of four children, 33 years and single)

Different modes of transport were used for food shopping by participants. Access to a reliable car, particularly a privately-owned car, was said to help the most and enabled access to larger supermarkets for food specials and buying food in bulk:

We didn’t have a car before but have one now. Made it easier to get around and do the shopping.

(Aboriginal and Torres Strait Islander mother of five children, 29 years and partnered)

I don’t have transport problems and can go to the places I want to shop. Usually follow the bargains and try to buy in bulk.

(Aboriginal and Torres Strait Islander mother of three, 38 years and single)

Participants who accessed public transport, particularly buses, found it difficult when travelling with small children. Using taxis was another option, though this was expensive particularly when funds were limited:

Hard to take the bus with a baby and a two year old to go shop or clinic.

(Aboriginal mother of two children, 25 years and partnered)

#### 3.3.2. Minor Influencing Factors

Social pressures, emotional wellbeing and housing featured in discussions of food security with at least one-third of the participants.

##### Don’t Want the Kids to Miss out

Four participants spoke in detail about their school-aged children needing money for entertainment and social occasions, of which put a strain on family income. They did not want their children “to go without” or miss out on social experiences that their children’s peers were perceived to have:

We have problems sometimes with having enough money …only when we have visitors or things the kids want to go to, like the [Darwin] Show. All the other kids going to the Show and our kids don’t want to miss out. It’s only fair for them, they only kids and should enjoy themselves.

(Aboriginal Grandmother of 10 grandchildren, 44 years and widowed)

Kid’s like to buy from the school shop [tuckshop] like the other kids. Sometimes I really don’t have enough money but, give them anyway. I don’t want other kids at school to think my kids are poor.

(Aboriginal mother of seven children, 26 years and single)

##### I Used to Get Sad a Lot

At least two thirds of participants openly discussed their feelings of how they felt emotionally in relation to food insecurity. Eleven participants expressed feelings of being stressed, down, sad, lonely and of frustration or feeling inadequate in being a good provider for their children. Two of the four participants receiving Centrelink payments referred to the stigma of shopping with a Basics Card and the feeling of frustration and ‘shame’ (shame is a term used by Aboriginal and Torres Strait Islander peoples as feeling embarrassed either about themselves or others. I.e. feeling shame because no money on the card to purchase groceries and others looking on. Or, feeling shame for someone else in a similar situation.) in having little control over managing their finances. These two participants also believed they did not need to be income managed and described feelings of public humiliation when not having enough money on the Basic card for groceries:

Real shame job [embarrassed] for me to go shop and find out don’t have enough money on the card [Basic card] to pay for groceries. Have to leave everything—trolley and all—with everyone watching. Make me real shame.

(Aboriginal mother of two children, 25 years and partnered)

Other participants raised and spoke about wellbeing related issues with relevance to food and money. In particular these were emotions of feeling down or sad due to relationship breakdowns and family stresses:

I used to get sad a lot and not able to look after the kids properly.

(Aboriginal mother of three children, 34 years and single)

##### The House Needs Fixing

Of the 30 participants, four were owner-occupiers with seven renting privately and nineteen renting through public housing. One-third of the participants in rental properties discussed problems with general home maintenance, specifically with kitchen maintenance including problems with window fly screens, kitchen benches, kitchen cupboards and stoves:

…There are no flyscreens on some of the windows and in others there are holes. The rats get in at night and sometimes [we] can see and hear them running in the house. Sometimes they run over us in our sleep!

(Aboriginal mother of four children, 34 years and partnered)

A number of participants expressed frustration in home maintenance:

…We’ve told him [owner] about the kitchen cupboards falling apart and other problems in the house. Just doesn’t seem to want to do anything about it….

(Aboriginal mother of two children, 29 years and partnered)

We can’t use the benches properly ‘cause the tiles are broken and dirty (bench top is tiled). The stove doesn’t work either”. We told housing [public housing authority] we have problems months ago, but they still haven’t come to fix them. All we do is wait and see what happens.

(Aboriginal mother of four children, 34 years and partnered)

One participant who owned their home, indicated that having adequate food storage space helped with always having food on hand:

…we buy frozen vegies as well, because they last longer and we have them on hand to put in our food [cooking]. Well that helps with us. So, having a freezer helps as well [with food storage].

(Aboriginal and Torres Strait Islander father of two children, 36 years and partnered)

Whereas a participant who did not have adequate cold food storage had to shop more frequently:

I have what I need in the house. [I] Need a freezer. That way can buy more meat and put away, instead of going to the shop every day to buy meat for dinner.

(Aboriginal mother of seven children, 26 years and single)

### 3.4. Impact on Food Selection

Within this theme are sub themes that encompass participants’ views regarding food affordability; relationship between food and health; and food behaviour in association with food insecurity.

#### 3.4.1. Not Everyone Can Afford to be Healthy

Many references to food and health were made by participants. In particular, the benefits of consuming home prepared meals rather than take-away meals perceived as high in fat and sugar. Four of the 30 participants spoke in-depth about fresh fruit and vegetables being ‘healthy’ foods and important in the prevention of illnesses such as type 2 diabetes. These foods though were considered by these participants as expensive and not always affordable when compared with other less healthy food options. At least half of the participants referred to the high cost of food influencing food choice.

An Aboriginal woman for example understood the relationship with food and good health, yet felt she was unable to put this knowledge into practice due to limited money and high food costs:

[I] Find it hard sometimes to eat healthy like have fruit and vegetables every day. Sometimes [it’s a] bit tight with money and [I] buy food that fills you up. Fruit doesn’t [fill you up] and it’s expensive. …. Always hear about why important to eat healthy to stop diseases like diabetes, but when you try to, it’s very expensive.

(Aboriginal mother of three children, 29 years and partnered)

We’re told to eat right, exercise and be healthy, but it’s hard when everything costs so much to be healthy. Not everyone can afford to be healthy.

(Torres Strait Islander mother of four children, 30 years and partnered)

One participant, where money for food was not considered an issue, spoke of not wanting her children to eat too much processed foods and have more natural foods in their diets:

I like my children to eat fresh food and foods that are not over processed. Also, processed foods tend to have a lot of sugar and that’s no good.

(Aboriginal mother of two children, 29 years and partnered)

#### 3.4.2. Something to Fill Our Bellies

Participants referred to compromising food quality for quantity to ensure that there was enough to eat at each meal. Most participants spoke of the importance of eating healthy food at meal times. However, for some this was not always feasible and most important to these participants was ensuring enough food to eat “to fill bellies”:

I make sure my kids are fed and don’t go without. Some of our meals are not that healthy, but at least we have something to fill our bellies.

(Aboriginal and Torres Strait Islander mother of three, 38 years and single)

We can afford food, but not always healthy food. Sometimes, have hamper [tinned corned beef] and rice with bread for dinner. It’s filling and the kids are not hungry.

(non-Indigenous mother of four children, 30 years and partnered)

Some participants referred to strategies used to ensure the family did not go without a meal:

…Usually try to buy in bulk and cook meals in bulk to freeze and use later. Therefore, make sure my daughter never goes without food.

(non-Indigenous mother of one, 28 years and single)

#### 3.4.3. Making the Meal Stretch

Most participants mentioned the use of low cost starchy foods, such as rice, pasta and bread to ‘fill children up between meals’ or add quantity to ‘bulk up’ meals when unexpected visitors joined in a meal or to use up leftover foods:

…If not enough food for each meal, cook more rice or have bread. This fills you up. Only time this happens is when we have unexpected visitors at dinnertime [evening meal] and we have to stretch the food so everyone has something”.

(Aboriginal mother of three children, 34 years and single)

I make sure kids always eat weet-bix [wheat biscuits breakfast cereal] in the morning before go to school. Have something at school from the shop [school tuckshop] and when they get home usually have bread with something on it. Boys eat a lot and bread is cheap and fills them up.

(Aboriginal mother of seven children, 26 years and single)

A male participant spoke of his family’s experience with using up left over food and filler foods to bulk up meals:

It’s sort of a standard way (having rice) of making the meal stretch. Not that having enough food is an issue. But when we have leftovers, it’s a, way of making sure we have enough.

(Aboriginal and Torres Strait Islander father of two, 36 years and partnered)

### 3.5. Coping Strategies

As a coping strategy, social support in the form of accessing extended family was the most prominent form of assistance sought by participants to prevent or help alleviate food insecurity.

#### 3.5.1. Live with Mum and Dad, They Help Out a Lot

Extended family provided the most common form of support and the types of support sought were mainly for money and food, but for some families it was assistance with looking after children. For four participants, assistance was sought regularly where others sought assistance only when there were additional demands placed on the household income. Running out of money and/or food were the most common reasons for accessing social support which usually occurred during ‘money tight’ times when the ‘big bills’ were due for payment. An Aboriginal woman with a family who lived with her parents, spoke of how this living arrangement assisted with expenses and provided support with looking after her children:

Sometimes have problems with money. Especially when the bills come in at once and don’t always have enough to buy food. My three kids and me live at home with my mum and dad. This makes it easier for when I run out of money. Mum and dad have money for food.

(Aboriginal mother of three children, 34 years and single)

Other participants shared their experiences with accessing family for assistance when experiencing difficulties and this support being reciprocated:

My partner has family here and if we don’t have food, or money for food, we go over to family’s place for dinner [evening meal]. Or if someone has money, we’ll lend money. Our home is open to family if we have food and someone wants something to eat or money. But I always make sure we have enough for ourselves first.

(non-Indigenous mother of four, 30 years and partnered)

We do have problems with food sometimes. Especially when we get big bills and there’s not enough money for food. Usually, go to my mum and dad to ask for money or food. Glad I have them. Don’t know where I would go otherwise for help.

(Aboriginal and Torres Strait Islander mother of five children, 29 years and partnered)

There were instances where participants who had limited or no social support found it difficult:

I am not from Darwin and don’t really have family here. My mother is visiting and I know some people from the community where I come from. Bit lonely sometimes.

(Aboriginal mother of seven children, 26 years and single)

For one participant however, a falling out with a family member led to this young mother of two to seek support elsewhere which was limited and resulted in food and money problems.

Four participants received support from family with household chores and looking after children:

We haven’t relied on family to help us out with feeding us, only with looking after the baby and other household chores when my wife was sick.

(Aboriginal father of one child, 38 years and partnered)

Most participants mentioned that living with immediate family members (parents or siblings) reduced the financial burden of expenses and assisted with raising of children. Almost one third of the participants lived with extended family and seemed to be in this arrangement for similar reasons. A participant shared her situation where she and her family had recently moved in with her parents:

We used to be in government housing, but now me and my partner earn too much and had to give up our house and find a private house to rent. But we can’t afford to pay private rent. Too much and won’t have much money left for food and other things we need. Me, my partner and the kids moved in with my mum and dad. That way we can save money to buy our own house.

(Aboriginal mother of three children, 29 years and partnered)

A mother of one, recently separated from her partner spoke of having to move in with her family to cope with expenses:

My ex [partner] moved out about 2 months ago and it was hard paying the rent and bills, so [I] decided to move out to Palmerston and be with my family. Too expensive living in Darwin. [I] Don’t know how other people like me can live there.

(non-Indigenous mother of one child, 28 years and single)

#### 3.5.2. We Don’t Have It as Bad as other Families

Most participants experiencing food insecurity expressed that others were in a worse situation than their own:

We don’t have it bad as some families. At least we always have something to eat, bills are paid and [have] petrol for the car”.

(Non-Indigenous mother of four children, 30 years and partnered)

“We are doing better than some other families. I know some have to ask for food vouchers [from Centrelink] to buy groceries.

(Aboriginal and Torres Strait Islander mother of three children, 38 years and single)

It makes you feel a bit easier to know that your situation is bad, but that someone else is worse off to make yourself feel better or make light of your current situation. I don’t know, but I think that it’s across the board [whole population].

(Aboriginal father of one child, 38 years and partnered)

## 4. Discussion

This study examined issues relating to food insecurity within a cohort of urban-based care-givers of Aboriginal and Torres Strait Islander children and found common features that contributed to household food security issues and mechanisms of coping. The most striking finding was that participants did not initially identify with being food insecure, although many participants’ experiences indicated otherwise. In general, participants accepted the situation of running out of money, food or both, and having to seek assistance from relatives as a normal experience. This finding has also been described in a study involving six Inuit communities of Nunavut, Canada which described an incongruence between perceived food security status and experiences in obtaining enough food to eat [7]. In contrast, another study undertaken within an Inuit population from Nunavut, found participants who reported food insecurity also reported regular use of community food programs to assist with alleviating hunger [2]. Unlike Chan et al.’s (2006) study, Ford et.al. (2012) recruited participants who were registered with food assistance programs and these participants may have shared characteristics with those considered by this study’s participants as “worse off” [2,7].

A second finding was that for most, food insecurity was experienced occasionally and usually when larger bills were due for payment. Food insecurity for some however was a chronic problem and seemed to be often due to an inadequate or irregular income. Two Australian studies have found that the Commonwealth Government New Start Allowance does not provide an adequate income for families, or anyone, to meet healthy living standards [22,23]. The authors did raise whether introducing an independent mechanism, similar to that of the Minimum Wage Panel that assesses its adequacy, to review and set the New Start Allowance level [23]. Participants that reported to have enough money to meet their needs, tended to be in paid employment. Secure employment and stable housing have been shown in other studies to be strongly associated with food security [2,4,5]. In contrast seasonal employment [5,8], unemployment and underemployment (the underemployment classification includes those workers that are highly skilled but work in low paid jobs; workers that are highly skilled but work in low skill jobs and part-time workers that would prefer to be full-time. This is different from unemployment in that the individual is working but isn’t working at their full capability. (Source: http://www.investopedia.com/terms/u/underemployment.asp)) [2,3,4,7,10,11] have been reported as problematic in ensuring a regular income to afford food and other expenses among those experiencing food insecurity. Noted in the findings, a small proportion of participants were income managed and shared mixed views of their experiences. These were, not having enough money to meet the family’s groceries requirements; feeling anxious wondering if there was enough money on the card for food; and finally, that it stopped the ‘humbugging’ from others wanting a loan. A study undertaken by Brimblecombe et al. (2010) in ten remote communities of the Northern Territory where income management had been introduced, investigated the impact of income management on store sales [24]. Stores sale data were reviewed over a 35-month period and included 18-months of data prior to the introduction of income management. Focusing on fruit and vegetable sales and turnover, Brimblecombe et al. (2010) found income management did not have an effect on store sales over the study period [24]. However, the Government stimulus payment between November 2008 to January 2009 did have a positive effect on fruit and vegetable sales [24].

In our study, participants with and without employment, referred to the cost of living as contributing to their food insecurity experiences. In some instances, participants purposely lived with extended family to mitigate potential food insecurity with the rising cost of living, even though they reported to earn an adequate income. Chan et al. also found the cost of living and cash flow among the ‘working poor’ to negatively impact food security in Nunavut communities [7]. A study undertaken in a United States urban centre found the cost of home rental was the single biggest factor identified among a group of young mothers as contributing to food insecurity [4]. In the current study, not only were high rent and large bills contributing factors to the high cost of living and food insecurity experiences, but issues with housing maintenance and inadequate kitchen facilities were also associated with experiences of food insecurity.

The experience amongst this study population of “money tight” due to the payment of large bills and general cost of living has also been reported by other researchers investigating food security experiences and influencing factors [4,5,8]. Participants in these studies were either low income earners or recipients of welfare (government payments) and received support through government and non-government food and nutrition assistance programs. Food insecurity occurred when money ‘ran out’ before the next pay period and food and nutrition assistance was accessed at these times to alleviate food insecurity over the short term. This contrasts to the findings of this study, where participants did not report to access food assistance programs.

Whilst participants dealt with intermittent food insecurity through various coping strategies, they did not appear to seek assistance from relevant agencies to alleviate food insecurity. Instead, strategies participants put in place during the “money tight” times were to delay payment of bills or undertake part payment of larger bills through staggered payments, or to cut back “luxury foods”, such as sweets, soft drinks and desserts. Similar coping strategies were reported by a study among a group of 90 food pantry users in Washington, USA, where coping strategies included putting off paying bills and using up leftover food, preparing food in bulk and freezing food for later use [11]. A study among Latino immigrant families in North Carolina, USA, also reported participants coped with times of food insecurity by reducing purchase of foods considered expensive, such as meats and fruits and unnecessary foods, such as ‘soft drinks, snacks and eating out’ [8].

The strategies employed to cope with “money tight” times in this study were seen to be both positive (such as limiting purchase of sweets and soft drinks) and negative (compromising quality for quantity) in respect to food behaviour and health. In other studies, shopping for specials, bulk-buying, cooking in bulk and freezing food portions are examples of other pragmatic responses [4,5,8,11] to food insecurity which were also employed by participants within this study. Negative responses to food insecurity, similar to that reported by this study, have also been reported by others including forgoing healthier food options and choosing cheaper less healthier foods, reducing meal size or going without to ensure children eat [4,5,6,8,11].

There is debate to whether the behavioural purchase of unhealthy foods in preference to healthy foods is driven by need, due to healthy food not being affordable [2,6,7,25] or by poor dietary habits, established food preferences and poor food purchasing knowledge [7,9]. Poor eating habits among high and low-income earners have been considered as being due to laziness [25] and time constraints [11,26]. In the current study, as similar to other studies, participants perceived healthier food to be more expensive to less healthy food and expressed frustrations at not always being able to afford healthier food options and bewilderment as to why unhealthier foods appeared cheaper. Other studies have reported the cost of healthy food options as a barrier to healthy eating and have commented that low income gave participants little option but to buy highly refined, energy dense foods that provide calories at less cost than low-calorie nutrient rich foods [4,7,15]. Similarly, a study undertaken within a low income urban Australian Aboriginal population, found participants understood what were healthy foods, but were not always able to afford these foods [13]. The same was reported for a study undertaken in an Aboriginal population in remote Australia where participants perceived healthy food to be unaffordable [17].

A fourth finding is participants’ concerns for their children’s needs often characterised their food behaviour responses to food insecurity. Hamelin and others (2002) also noted experiences of anxiety by some participants in ensuring enough food for the children and the accompanying feelings of despair [10]. Upon similar lines, within this study several participants expressed concerns for the acceptance and social inclusion of their children by peers and how this exacerbated the risk of food insecurity due to allocation of limited food money to non-food items or entertainment. Participants also spoke of the experiences of others’ they knew, specifically with drug and alcohol use and how this impacted on families’ food security situation. Temple’s (2018) study looking at the association between stressful events and food insecurity in Australia, found between the food secure and food insecure respondents there was a prevalence greater than 10% that included among other stressors, drug or alcohol related problems [27].

Use of a private vehicle, as reported within the current study to access food outlets, enabled people to seek out food bargains and specials. Specifically, access to a private vehicle was advantageous in accessing larger supermarkets where food was often cheaper and of more variety. This was also noted by other studies where public transport (buses and taxis) were considered by participants as unreliable, inconvenient or expensive and therefore, found to negatively impact on food security [4,5,9,28,29].

Finally, unlike the variety of social support systems accessed by other populations experiencing food insecurity, including food assistance programs, food charity organisations, faith communities, neighbours and friends [2,3,4,5,6,7,8,9,10,11,26], this study is unique in that family support was the only resource reported to be accessed for assistance. Other studies have also identified the extended family as a social support system [2,3,4,8,9,11] and identified support from friends and neighbours as a main coping strategy in response to food insecurity [5]. Only one study however, mentioned living with family as a temporary measure [2]. Residing with family members, particularly parents, was mentioned within the current study as a way for families to cope with living expenses. For some, this arrangement also provided support with child care. However, we did not the food security of all family members and it is possible that other members have influence perceptions of food security e.g., food insecure members moving in with food secure persons may heighten the odds of the later experiencing food insecurity.

Central to the study participants’ social support system, was the action of reciprocity where families coped through inter-reliance on each other for food, money and other necessities. For instance, participants reported that when they had food, they would provide for other extended family members. Then when they would ‘run out’, extended family assisted in return. Reciprocity was also mentioned in a study, where young mothers would rely on family members for assistance with food and then ‘return the favour’ when family members experienced difficulties [4].

Within this study, as with other literature, reciprocity forms a cultural practice of sharing among Aboriginal and Torres Strait Islander peoples that is important in maintaining and reinforcing cultural social bonds with individual and group relationships [14]. As noted by Chan et al. and Ford et al., reciprocity has a place in maintaining and reinforcing family and broader community relationship obligations as well as cultural identity and practice among Inuit [2,7]. Among the Inuit these sharing networks extended to hunted traditional foods where excess was provided to the more vulnerable members of the community who cannot obtain these foods [2,7]. For those who could not hunt, money or other services were exchanged for traditional foods to keep with continuing cultural practices [2,7]. This concept of sharing traditional foods in a reciprocated environment to help each other out is also evident in this study where food and monetary assistance was sought and provided within families.

Discussions with participants within our cohort identified reciprocation as an expectation, and a given cultural practice to maintain family relationships. Such sharing support structures however are also fragile and relationship upsets can result in limited or no support as experienced by one participant in this study. In contrast, a food insecurity study undertaken in an Aboriginal and Torres Strait Islander population living in Victoria, indicated that accessing family and friends for assistance was not reported by this population [13]. External programs providing assistance in the forms of food vouchers, as well as charity organisations providing meals and food parcels, are available within the study location. However, these services were not mentioned by participants as being accessed for assistance. This finding however should be interpreted with caution as participants may have chosen not to share this information and seeking knowledge about access to such services was not the purpose of our study.

Finally, unlike studies [4,5] indicating the importance of furthering education to gain employment or improve opportunities for higher paid work as a long-term solution to overcoming food insecurity, this was not found within our cohort. A possible reason is, as identified in this cohort being food insecure is normal i.e., ‘normalisation of a pathology’. When problems with food security are encountered, reciprocated arrangements with family as a coping strategy provide an immediate solution and reinforce traditional Aboriginal and Torres Strait Islander relationships. Therefore, furthering education or skill development for employment as an option to alleviate food insecurity may not be considered by participants in our cohort. This is an important issue and further understanding of what constitute food security will be useful in the future.

## 5. Conclusions

We found that Aboriginal and Torres Strait Islander families in our cohort had varying direct experiences with household food insecurity. A major contributor to this is their limited financial resources in conjunction with rising living costs. For the majority, this was intermittent and occurred when the larger bills were due for payment. For some, however, food insecurity is a chronic problem where expenses outweighed income. Not having enough money to buy food and take care of living expenses is a universal experience for those on limited incomes. Similarly, for the participants in this study having a limited income impacted on their circumstances and other factors also impacted on food security, including transport and concern for social image.

We also found that the extended family was the major form of support for assistance and played possibly a broader cultural role in sharing as also identified among Inuit populations [2,7]. This was also a reciprocated arrangement where families would help each other out. However, it could also be considered fragile as support was very reliant on harmonious relationships between family members and may be considered only functional when relationships are.

### 5.1. Strengths and Limitations

Unlike other similar published research, a strength of this study is participant sampling in that recruitment was not undertaken through food assistance programs. This study therefore, provides a broader view of food security experiences from a perspective where people are either experiencing food insecurity or not. As previously referred to, this qualitative study is one part of a larger study. Although the sample size is small, initial discussions followed by in-depth interviews consolidated themes, as a point was reached during data collection where no new information was forthcoming and data saturation was reached. The majority of participants were however, from well-established families within the two study locations. Therefore, the findings are more applicable to families who are long term residents of Darwin and Palmerston with extended family networks. Caution is required in generalising findings to all families in the Darwin and Palmerston regions and other similar populations. There are also possibilities of bias with findings reflecting the views of one gender more so than the other. Future studies may need to consider recruitment and sampling strategies that address gender balance.

The interviews were undertaken by an Aboriginal Public Health Nutritionist which was positive in communicating and establishing a trusting relationship with participants of which was captured within the interviews. The study design and methodology could be considered for future qualitative research investigating unexplored topics to generate new knowledge in learning more about Aboriginal and Torres Strait Islander peoples’ understandings and experiences of food security. Finally, this qualitative research has unveiled ‘new’ understandings of food insecurity experiences and coping strategies from an urban Aboriginal and Torres Strait Islander population perspective that otherwise, may have remained unknown to the broader community.

### 5.2. Implications

A possible solution to assist with meeting payment of expenses is support to families to set up direct debit options of smaller regular payments to offset larger bills and undue financial pressure.

Transport, preferably access to a private car, was also deemed essential by some to undertake food shopping. There could be possible scope for services and other assistance programs to consider these needs. For instance, food shopping assistance for older Australians and the disabled is provided through government and non-government services [28]. Major supermarket chains in Australia such as Coles and Woolworths provide an online shopping and delivery service for a fee. This may not be available by all stores and may not appeal to all consumers. However, it could be considered by government and non-government services to provide food shopping assistance, including a subsidised or free food shopping delivery service, for low income families with young children.

There is a widely held perception that the cost of healthy foods makes a healthy diet unaffordable for families. Participants of this study referred specifically to the cost of fresh fruit and vegetables and the importance of these in prevention of chronic disease, such as type 2 diabetes. Consideration of economic access to healthy foods in public policy seems critical for improved health outcomes. A potential solution may involve food subsidies or similar. There are also opportunities for local councils to consider availability of public allotments to encourage community or family group food gardening to supplement diets though, this was not an option identified by study participants. The perception of fresh fruit and vegetables being costly is worth further research investigation, particularly in assessing the affordability of healthy foods within the study location.

In ensuring appropriate and sustainable safety nets that provide assistance to families, it is important to acknowledge the existence of support services accessed by families that are not recognised within the mainstream and are specific to Indigenous Australians. These include positive family associations. Potential scope for current services is to consider an approach in connecting with family networks for provision of support services, such as financial counselling. Such services have potential to provide peer support family counselling where members experiencing difficulties are supported by family member(s) to engage with services and work through issues.

This study has clearly identified food insecurity experiences among the study population to be related to monetary expenditure outweighing income, particularly with the payment of larger bills. Being in a situation where money is limited and expenses out way available funds, fulfilling a family’s social, cultural and physical needs requires a fragile balance of continually adjusting food access and purchasing behavior at time when ‘money is tight’, maintaining family support structures, and upholding social status.

## Figures and Tables

**Table 1:** Demographic characteristics of households.

Characteristic		Initial Discussions (*N* = 30)	In-Depth Interviews (*N* = 6)
Parent gender	Female	27	4
Marital Status	Partnered	17	5
Indigenous status	Aboriginal	19	5
	Torres Strait Islander	1	0
	Aboriginal and Torres Strait Islander	6	1
	Non-Indigenous Australian	4	0
Care giver	Parent (mother/father)	27	6
	Other (grandmother/foster carer)	3	0
Parent age (yrs)	Median (range)	44.5 (17–58)	35 (25–39)
Residents in house	Median (range)	6 (3–15)	5.5 (3–10)
Number of children by age group (*N* = 57)	6 to 24 months	19	3
	25 to 48 months	30	5

## Appendix A

**Table A1:** Inductive development processes towards key guiding questions for the in-depth qualitative interview.

Theme	Notes	Guiding Questions
Influencing factors		
Income quarantining (Basic Card)	Limited control over own money. Anxious when going shopping, as don’t know how much money available on basic card for spending. Feelings of shame/embarrassment/anger when: - can’t pay for it (not enough money on card) and don’t have extra cash. - On the scheme and have no say in being on it or not.	Tell me more about your experiences with the Basic Card. Why do you like/dislike being Income managed? How does this make you feel?
Housing problems (more around maintenance)	House needs fixing, takes a long time before something is done, participant has little control over the situation. Limited availability of public housing, hard to find a place to live. Sense of feeling powerless, beyond people’s control	Tell me about your house. Is everything good? i.e., windows, benches, etc.? Does anything need to be fixed?
Food preparation and cooking facilities	Food storage, preparation and cooking facilities: - Need for working stove - Need for a freezer	Do you like to cook? Can you tell me about your experiences with cooking (good/ok). What stops you from cooking?
Money problems	About not having enough money to fulfil own and families’ needs/wants/requirements. Impact of the cost of living in Darwin and Palmerston—everything is expensive. Limited money, prioritise what spending on Always make sure the children are fed, don’t go without.	Do you have enough money for what you need? If no money problems/worries, what do you do to make sure everything is good? Can you tell me about your money problems/worries? How often do you have money problems/worries? Are money problems ongoing (all the time)? When you have money problems, how do you prioritise spending? Are there things that do you do to cope with the problem?
Social Inclusion	Not wanting money problems to impact on children’s lives to point where excluded from social events, outings, what their peers have, etc.	Is it important to you and your kids that you don’t miss out on what other families have? What are some of the things you do to make sure you and your kids don’t miss out on having what others have?
Budgeting	Always make sure money for food, even if not healthy. An already tight budget for food and regular expenses. Additional expenses puts a strain on the budget therefore, spend less on food and tend to eat less healthy.	How do you make sure there is enough money for things you/your family need between paydays? Does this always work, or do you sometimes find it hard? What else do you do to try and make it work?
Filler Foods	Low cost, high calorie foods to stretch meals and ‘fill you up’—bread and rice. E.g of filler foods for a meal—cheap tinned meats (hamper) and rice with bread.	Do you have enough food at each meal for everybody? What do you do to make sure everyone has enough food? Tell me about these foods (filler foods) and the reasons you choose them? - Cheaper, feed more people (stretch meals) and fill you up. - Comfort, familiar food
Wellbeing	2 participants talked about feeling sad. As mentioned previously, feelings of shame, embarrassment, anger, anxiety, powerless.	***If possible, find out more if these feelings are related to money worries/problems.*** How do you feel when you don’t have enough money? Do you think about having enough money a lot?
Other’s worse off	Acknowledge other families experiences similar problems and probably more worse off. However, also put this down to possible use of drug and alcohol and this is where money is diverted to.	From what you know, do you think your family ‘has it hard’ compared to other families?
Transport	Few transport issues to go places to shop, particularly when relying on public transport and travelling with small children	What are your experiences with having regular transport to go shopping?
Social problems	Identify money (income) diverted to social problems such as drug, alcohol and gambling impact on having enough food.	What are your thoughts on why some families may experience problems with having enough food?
Food Shopping	Transport and the amount of shopping undertaken impacts on where people shop. Others that ‘plan’ where they shop according to where bargains are—buy bulk.	Do you worry about going food shopping (regularly)? Do you plan where you will shop and what you will buy before you go? Are you able to go to the shops when you want to?
Coping Strategy		
Support Networks	Families rely on extended family and friends’ networks for support and ‘fill the gaps’	Tell me more about the support you have when you are having difficulties? Do you think you rely on this support network? Where would you go if you didn’t have this support network?
